# Cultural Differences in Perceptual Reorganization in US and Pirahã Adults

**DOI:** 10.1371/journal.pone.0110225

**Published:** 2014-11-20

**Authors:** Jennifer M. D. Yoon, Nathan Witthoft, Jonathan Winawer, Michael C. Frank, Daniel L. Everett, Edward Gibson

**Affiliations:** 1 Department of Psychology, New York University, New York, NY, United States of America; 2 Department of Psychology, Stanford University, Stanford, CA, United States of America; 3 Brain and Cognitive Sciences, MIT, Cambridge, MA, United States of America; 4 Department of Sociology, Bentley University, Waltham, MA, United States of America; Ecole Polytechnique Federale de Lausanne, Switzerland

## Abstract

Visual illusions and other perceptual phenomena can be used as tools to uncover the otherwise hidden constructive processes that give rise to perception. Although many perceptual processes are assumed to be universal, variable susceptibility to certain illusions and perceptual effects across populations suggests a role for factors that vary culturally. One striking phenomenon is seen with two-tone images—photos reduced to two tones: black and white. Deficient recognition is observed in young children under conditions that trigger automatic recognition in adults. Here we show a similar lack of cue-triggered perceptual reorganization in the Pirahã, a hunter-gatherer tribe with limited exposure to modern visual media, suggesting such recognition is experience- and culture-specific.

## Introduction

A core principle of vision science is that perception is not simply a passive reflection of the external world, but a process of constructive interpretation of inherently ambiguous input. Consider a shadow projected onto a wall. The same silhouette can be created by different objects of different sizes at different distances from the viewer. Images projected onto the retina have the same inherent ambiguity, and a wide range of perceptual judgments ranging from lightness [1], to color, to depth, to shape and identity, are the result of “unconscious inferences” by the visual system [2]. Such inferences are often presumed to be automatic and culturally universal [3]–[5].

**Figure 1:**
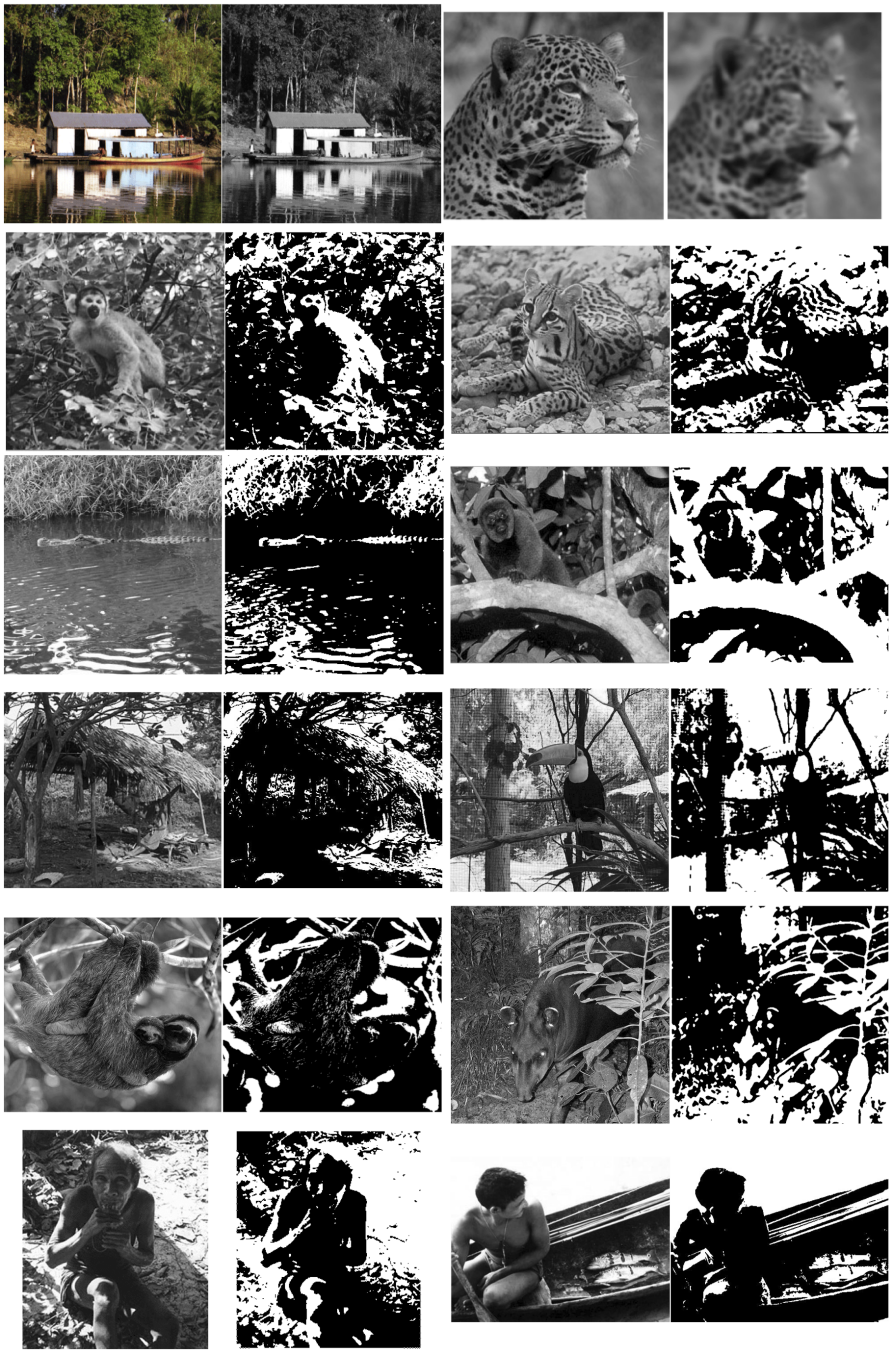
All stimuli used in the experiment. Row 1 shows warm-up items with simpler transformations: houseboat and jaguar. Rows 2–6 show test items. Left column: squirrel monkey, alligator, woman in hut, sloths, older man. Right column: ocelot, howler monkey, toucan, tapir, fisherman. Cue items are shown to the left of test items. For full size stimuli, see Figures S1–S12 in File S1 in order to recreate viewing conditions under which recognition is trivial for western adults.

The interpretative processes that give rise to a coherent percept or “gestalt” often occur effortlessly and without awareness, but they can be made explicit by examining images that are not correctly interpreted upon initial viewing, such as the famous two-tone depiction of the Dalmatian in the snow [6] or the two-tone ocelot in Figure 1 (right column, second row). People often fail to recognize the two-tone image; when shown the corresponding photograph, however, they find the two-tone often transforms suddenly into a coherent percept. Observers viewing the ocelot in the two-tone will often make figure-ground errors, incorrectly assigning some background regions to the figure, some figure regions to the background. Reconfiguring figure-ground assignments after viewing the photograph is to “reorganize” one's initial grouping to achieve a different perceptual state [7]. If the viewer ultimately recognizes the previously unrecognized image, perception reorganization is said to have been successful. (Following the Gestalt school, we use the terms “perceptual organization” and “perceptual reorganization” to emphasize the process by which local image features are appropriately integrated or segregated in order to arrive at a meaningful interpretation of the image—a “gestalt” [4]).

Not all two-tone images require perceptual reorganization through extrinsic cueing to be recognized. Many logos are designed to be easily recognized while consisting of only two tones (e.g., the World Wildlife Federation's panda logo), and simple black and white line drawings are similarly recognizable on initial presentation. It is nonetheless the case that one can create two-tone images that challenge the perceptual system and for this special type of two-tone image, presentation of the corresponding photo cue readily triggers perceptual reorganization [8]–[10].

Many of the principles underlying perceptual organization are thought to be universal, based on demonstrations of sensitivity to these principles even in very young infants and remote cultures [5], [11]. There is also a body of evidence reporting variable susceptibility to certain illusions and other perceptual phenomena across different populations. These results suggest an important role for culturally variable factors, including experience with artifacts such as photographs [12] and digital clocks [13], culture-specific processing biases [14], and exposure to urban versus rural vistas [15]; for review of older work, see [16].

Culturally invariant mechanisms of development such as the physiological maturation of the visual system predict differences in perception between children and adults. But children may also become more strongly enculturated into the practices of perceptual inference and interpretation accepted in their particular community over time, similarly predicting differences in how children and adults perceive the world [17]. One particularly striking phenomenon in perceptual development is the deficient recognition of two-tone images in young children under conditions that trigger automatic and effortless recognition in adults [7], [18]. When faced with images like Figure 1 (even ones containing familiar creatures), children—like adults—often struggle to recognize the animal. But unlike the adults, children have very significant difficulty recognizing the animal even when the two-tone image is placed side by side with the original picture.

One issue that is left unaddressed by these developmental data is whether the rapid perceptual reorganization reported by adults is a necessary consequence of having a mature visual system, or whether it is the result of knowledge and experience acquired in a specific cultural context. To address this question, we turned to a population with very different visual experience than the participants in typical perception experiments: the Pirahã. The Pirahã are a hunter-gatherer tribe inhabiting a remote region of the Amazon. They are of particular interest for our current study because of their limited contact with modern visual media and their sparse material culture [19]. Like young children in a modern industrial culture, Pirahã adults have little experience or knowledge of the visual transformation that links a photo and two-tone image. But unlike children, they possess both physiologically mature visual systems and a lifetime of experience with complex visual tasks such as hunting and fishing.

We tested Pirahã adults and English-speaking controls on their ability to recognize two-tone images given the corresponding photographs as cues (Figure 1). We predicted that, like children and U.S adults, the Pirahã would have difficulty recognizing two-tone images uncued, that is without seeing the accompanying photo. If expertise in interpreting symbolic visual materials is a key factor in cue-triggered two-tone reorganization, then the Pirahã—like children but unlike U.S. adults—would have trouble recognizing the cued image even in the presence of the photo.

## Methods

Participants included adult members of the Pirahã tribe (n = 9, mean estimated age  = 30 y) and as controls tested with the same stimuli, Stanford University students, faculty, and staff (n = 8, mean age  = 26 y). An additional control task with additional stimuli was tested on Stanford students (n = 10, mean age  = 19 y). The visual acuity of the Pirahã population was tested by DE and others some years earlier as part of a basic screen for medical services; the population was on the whole normal, with no cataracts and a small incidence of nearsightedness.

*Ethics statement*: All US participants gave written consent to participate in this research, and the consent procedure and study were approved by the Stanford University Institutional Review Board. Written consent forms were stored in a secure location as required by the Stanford Institutional Review Board. Experimental research with Pirahã participants was approved by the University of Manchester Committee on the Ethics of Research on Human Beings. This committee authorized a waiver of written consent because the Pirahã participants were not able to read or write. Participants gave oral consent and research goals were explained (as well as possible, given both the linguistic and conceptual vocabulary available) by DLE. The oral consent of all Piraha participants was communicated by DLE to JMY and recorded in a laboratory notebook.

Ten two-tone images were created in Photoshop by blurring and posterizing (reducing the number of distinct gray scale values, in this case to two: black and white) grayscale photographs of animals and individuals found in the Pirahã participants' everyday environment (Figure 1). The amount of blur and the black/white threshold points were set independently for each photograph based on a repeated trial and error procedure until we were satisfied with the subjective impressions that the two-tone was (a) hard to recognize without first seeing the photograph from which it was derived (“uncued”) and (b) easy to see after seeing the photograph (“cued”). This stimulus creation and selection were guided by the perceptual judgment of the experimenters. Images were printed onto 12×12 cm cards.

These two-tones are similar in appearance, but different in method of stimulus creation (as well as experimental purpose) from the stimuli known as “Mooney faces.” Mooney himself used the stimuli to study “closure”-based recognition of individual images, analogous to our “stage 1 uncued” presentation. Mooney's faces were hand-drawn artist's renderings of human faces under extreme illumination conditions [20], so there is no corresponding photo from which the images were derived.

**Figure 2:**
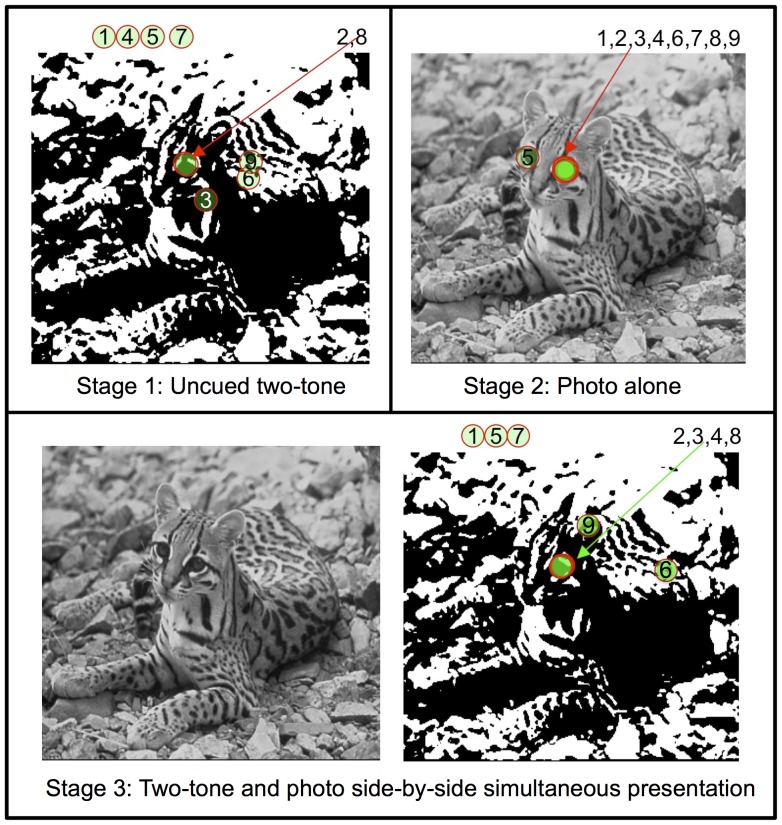
Upper left: an example two-tone stimulus from the Pirahã study. Subjects were first presented the two-tone alone and asked to point to the location of an eye or person in the image. Red circles mark where Pirahã participants indicated an eye, and numbers indicate individual participants. Circles outside the image show responses of the form “there are no eyes here.” Only two participants (2,8) correctly pointed to an eye in this image during Stage 1. Upper right: Performance of Pirahã participants on the original photo, which was presented alone after the two-tone image was removed from view. All participants correctly pointed to one of the two eyes. Bottom row: performance of Pirahã participants on the two-tone image during Stage 3, when it was shown side-by-side with the photo. Two Pirahã participants succeeded uncued (2 & 8), two more succeeded with the photo present, indicating reorganization (3 & 4), and five did not show evidence of photo-triggered perceptual reorganization (1, 5, 6, 7, 9).

Two other image pairs were tested which did not include two-tones and for which the correspondence was easier to see (Figure 2, top row, two image pairs). These served as warm-up items and to ensure participants understood the task.

Each trial proceeded in three stages. In stage 1, participants were shown a two-tone image and asked to indicate their recognition by pointing to the location of the eye or Pirahã person in the picture (Figure 2). Responses were marked by placing a sticker at the indicated locations. Trials in which the target was not initially identified were considered “candidate reorganization trials.” These trials were of particular interest as they provided a test of whether an initially unrecognized two-tone image could be successfully reinterpreted after seeing the corresponding photo. These trials proceeded to stages 2 and 3. In stage 2, participants were shown the corresponding photograph alone and asked to point to the location of the eye or Pirahã person. In stage 3, the two-tone image and photograph were shown side-by-side. The experimenter then pointed back and forth between the two images using the Pirahã word for “same” to convey the correspondence between photo and two-tone. After this instruction, the subject was again asked to point to the location of the eyes or person in the two-tone image.

Viewing distance for Pirahã participants ranged from about 1.5 to 3 ft and was not precisely controlled. Variability in this range is unlikely to affect recognition. In a separate control study to test for the possibility that close viewing interfered with perceptual re-organization, U.S. adults viewed two-tones from a much closer viewing distance than seen in any participants (9 in.) and performed at ceiling on candidate reorganization trials (100%). In addition, U.S. preschoolers, a similarly low reorganization population, viewed two-tones from distances of 2 and 4 feet with no difference in performance [21].

We additionally tested Stanford students on an alignment manipulation task. This task controlled for the possibility that U.S. participants' performance on the task was not due to recognizing the two-tone images, but merely to locating the point on the two-tone card in the same location as the corresponding point in the photograph. This study was identical to the main study, except that the images were cropped by 10% on two adjacent sides (e.g., top and left), chosen at random, with the constraint that the corresponding two-tone and photo were not cropped on the same two sides. Thus the eye or head was in a different location on the printed card in the photo and in the two-tone. If US participants were solving the task by pointing to the same location on the cards rather than by identifying the image features in the two-tone image, they would not have successfully located the eye in the two-tone image in this experiment.

## Results

**Figure 3:**
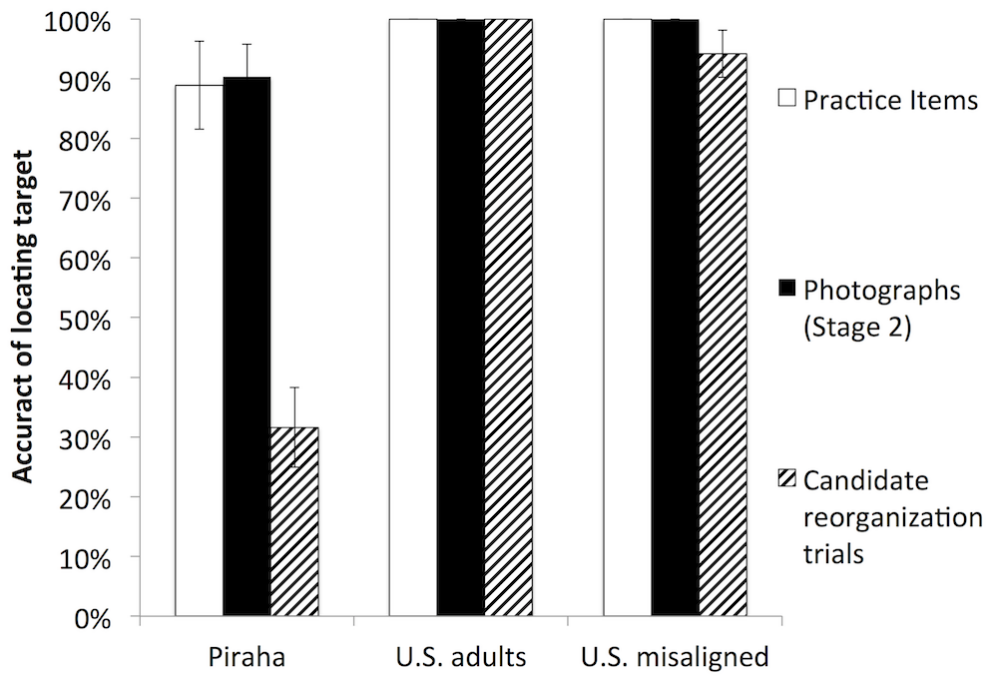
Summary of results from the Pirahã and the two U.S. control groups. Bars show participants' accuracy on photographs, practice items, and candidate reorganization trials (those trials on which the two-tone image was not recognized uncued). Error bars show the standard error of the mean.

Pirahã participants and U.S. control participants on the same task successfully indicated the target locations (either eye or person) on the non-two-tone practice images without the corresponding photo cue (controls 100%, Pirahã 88.9%), showing participants understood the task (Figure 3, white bars). Controls located the targets successfully in uncued two-tone images on 72.5% of trials. Initial recognition in Pirahã participants was less frequent (22.5% of trials). Controls identified the targets in the corresponding, untransformed photos 100% of the time and the Pirahã 90.3% of the time (Figure 3, black bars). All Pirahã participants correctly indicated the target on at least 7 of the 10 photos. Data from trials where the Pirahã did not correctly recognize the photo were excluded from subsequent analysis.

The trials of primary interest were candidate reorganization trials: trials on which participants did not initially locate the target in the two-tone (incorrect Stage 1), but did locate it in the photo cue image when it was presented alone (correct Stage 2). We assessed performance on only these trials (% correct Stage 3) by calculating the percentage of two-tones recognized after viewing the photo cue and dividing by the total number of candidate reorganization trials. U.S. control participants consistently showed cue-driven perceptual reorganization, always (100%) correctly indicating the eye or the Pirahã person on previously unrecognized two-tones. In contrast, Pirahã participants succeeded on candidate reorganization trials only 31.6% of the time. Two Pirahã participants never demonstrated perceptual reorganization, and the highest rate of reorganization for any Pirahã individual was 60%.

Control participants in the misaligned condition—like the controls in the main experiment but unlike Pirahã participants—showed near perfect performance on candidate reorganization trials (94.2%). This result would be expected if control participants experienced reorganization, and their performance did not depend solely on a spatial alignment strategy to localize features.

To summarize these observations statistically, we conducted a repeated measures ANOVA with a 3-level within-subject factor (trial type: practice items, photos, candidate reorganization trials) and a 3-level between subject factor (group: Pirahã, US controls, US misaligned condition). There was a main effect of group (*F*(2,23) = 32.6, η^2^ = 0.74, *p*<0.001) and a trial type x group interaction (*F*(4,48) = 8.35, η^2^ = 0.41, *p*<0.001). Pairwise comparisons reveal that the Pirahã differ from both US groups (*p*s<0.001), who do not differ from each other (Bonferroni corrected). Similarly, accuracy on candidate reorganization trials differs from accuracy on practice trials and photo recognition (*p*s<0.001), which do not differ from each other. A follow-up *t*-test compared Pirahã candidate reorganization performance to US control performance in the misaligned condition (when US controls do not have access to a non-recognition-based location matching strategy), showing that Pirahã performance was significantly lower (*t*(17) = 8.26, *p*<0.001).

We include additional analyses in the discussion related to different possible interpretations of Pirahã performance. Raw data are available in Dataset S1.

## Discussion

We tested whether Pirahã participants showed perceptual reorganization of two-tone images when they were viewed side-by-side with the original (cue) photograph from which they were generated. Although U.S. control participants performed at ceiling, successfully identifying the target location in every previously unrecognized image, the Pirahã found this task extremely challenging. The relative lack of cue-driven perceptual reorganization in the Pirahã is especially striking in contrast to the reported ease, vividness, and automaticity of reorganization in the control group, which persisted in the face of spatial misalignment.

But why is reorganization so much less frequent in the Pirahã group? We begin by discussing candidate hypotheses relating to strategic choices, task interpretation, stimulus familiarity, and task difficulty, and then discuss possible conceptual or experiential sources of differences in perceptual reorganization.

**Figure 4:**
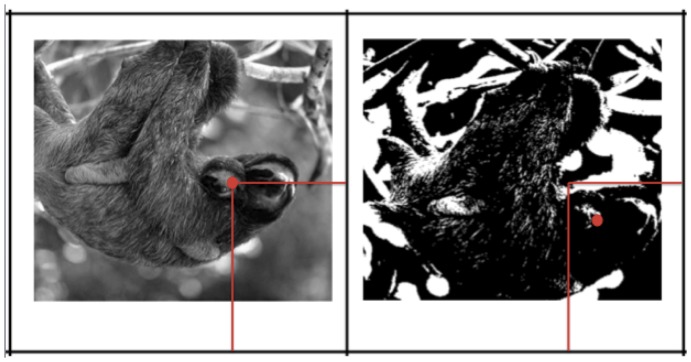
Example of misaligned photo and two-tone image pair. This image shows the actual degree of misalignment, but participants were never informed about the degree or direction of misalignment or even if any misalignment or distortion occurred between the images. The intersection of the horizontal and vertical red lines shows the same geometric point relative to the frame of the images. Participants could identify these matching points even without reference to the underlying images (for example, if the cards were blank). Actual corresponding features are shown with the red dot, and were displaced 1.8 cm in a direction that varied from image pair to image pair.

First, we take up the issue of whether possible strategic differences rather than recognition differences between the Pirahã and US groups can explain our findings. One proposal is that the US and Pirahã may have the same levels of cued two-tone perceptual reorganization, around 30%. However, US participants are accurate on the remaining 70% of trials because of some non-perceptual strategy they use that does not depend on recognizing anything in the two-tone image. We can rule out one version of this account, based on the results of the US misaligned condition. US adults are just as accurate at finding a corresponding feature between the photo and two-tone images when the two images no longer share a predictable coordinate frame relative to one another (e.g. as in Figure 4). Thus, they would not be able to rely on simple matching of spatial coordinates relative to the image frame to find the corresponding location in the photo and two-tone image pairs. Instead, some level of perceptual reorganization was required in order to identify the unpredictably displaced location in the two-tone image within the bounds of the recognized figure. Nevertheless, some weaker version of this general hypothesis is similar to what we believe to be true. In fact, the “perceptual literacy” hypothesis we describe below specifically attributes Pirahã and US performance differences to cultural differences in training and education with visual symbolic materials.

Second, our data are inconsistent with an account driven purely by differences in task interpretation between the Pirahã and the U.S. controls. Good performance on the practice trials and on the photos themselves demonstrates that the Pirahã understood the general task instructions. The experimenters made their best effort, both verbally and gesturally, to explicitly indicate that the photo and two-tone images corresponded and were “the same.” Even if Pirahã participants did not know how to interpret the experimenter's instructions initially, they would presumably understand that the image pairs corresponded after a successful spontaneous reorganization. However, success on one candidate reorganization trial did not lead to reliable gains in accuracy on subsequent trials (41% accuracy including only trials after first successful reorganization vs. 32% across all trials, two sample *t*-test, *p* = 0.4), suggesting that the experience of reorganization did not substantially change the overall difficulty of the task.

Pirahã participants did sometimes respond “no eyes/person” when asked to find these targets on the two-tone images. One might worry that the Pirahã do experience cued perceptual reorganization similar to US adults – but they interpret the task instruction of pointing to the target location differently from the US participants. Perhaps they have a stricter criterion for responding, “yes, here is the eye/person”, and this is reflected in their “no eye/person” responses. However, they gave this response in only 37 of 90 trials, and 27 of the 37 occurred after they had already made an accurate response by locating eyes or people in previous two-tones. In addition, inspection of the specific items reveals that each of the two-tone items was related to a “yes” response across at least two and as many as eight of the nine participants. In other words, although items in our stimulus set varied in the specific details of their appearance, no item was judged by the Pirahã group to be completely featureless. These items included both images that had clearly distinct eye-spots (e.g., squirrel monkey) and no distinct eye-spot (e.g., fisherman). It is therefore more parsimonious to interpret the occasional “no eye/person” responses as indicating lack of recognition as opposed to a different interpretation of the task instructions.

Third, since the photographs depicted people and animals in their environment, it is also unlikely that the result is due to a lack of familiarity with the pictured items. In fact, the particular animals and people depicted in the photographs were more familiar to the Pirahã than the controls (one of the pictures contained a person known to members of the tribe, and several of the Pirahã participants spontaneously produced his name on seeing the photo).

Two-tone image recognition can fail at multiple stages by different mechanisms. A fourth, possibility is that poorer performance among Pirahã participants in the candidate reorganization trials was not due to a difficulty in using a photograph to reinterpret a two-tone (presumably a more top-down process), but rather a difficulty in recognizing the *uncued* two-tones, or simply a difficulty with any hard task. Recognition could have failed at a coarser level than photo and two-tone comparison—perhaps during early bottom-up stages of processing like basic perceptual organization, due to overall greater difficulty. Indeed, the Pirahã participants had significantly lower accuracy at two-tone recognition in the un-cued condition, indicating that two-tones are more difficult for them to see. To address whether this also explains why they were less likely to perceptually re-organize after seeing the photo, we compared items of similar difficulty in U.S. and Pirahã groups.

There is currently no theory to predict what types of two-tones will be reorganized more “vividly” or “completely” or “automatically” than others. In the absence of such a theory, we define difficult two-tones behaviorally, as those that are rarely recognized uncued; similarly, easy two-tones are those that are recognized frequently even when they are uncued. In pilot testing with a larger set of stimuli (40) on U.S. adults (n = 9), successful *reorganization* occurred consistently regardless of variation in uncued performance – two-tone “difficulty.” Even for the 5 stimuli with the poorest uncued recognition (46% success), participants successfully re-organized on nearly all trials (93%), far more often than the Pirahã in the main experiment reported here. In contrast, considering only the two “easiest” stimuli with the highest uncued recognition by the Pirahã (78% success), Pirahã participants who did not initially recognize these stimuli still re-organized only infrequently (25%, 1 of 4 trials). Assuming a binomial distribution based on the reorganization probability of English speaking participants (93%), the likelihood of the Pirahã data—reorganizing only once or fewer out of 4 trials—is less than one percent. Thus, we do not believe that difficulty in recognizing the two-tones without a cue can explain our findings.

How then should we interpret the striking failure to reorganize the two-tones in the Pirahã participants? Our data indicate that a mature visual system is insufficient to guarantee good performance on this task. It is possible that the similarly low rates of photo-triggered reorganization of two-tone images in young U.S. children [7], [18] and Pirahã adults are unrelated. But the existence of reduced reorganization in an adult population opens up the possibility that developmental failures in perceptual reorganization in young U.S. children may also be explained by a mechanism distinct from visual system maturation. Thus, one possibility is that the results reported here, together with the previous studies on young children [7], [18], [21], [22], reflect a role for expertise with visual symbolic materials (writing, art, photos, etc) in assisting with reorganization.

Visual symbolic materials are notably sparse in the Pirahã community, but ubiquitous in the US and other industrialized cultures. A provocative interpretation of the current data is that cultures with ubiquitous visual symbolic materials may entrain a figurative “perceptual literacy,” analogous to reading literacy. Such literacy would consist of a body of perceptual and cognitive skills that together create expertise in decoding such materials. What kinds of perceptual and cognitive skills might be included in such visual symbolic expertise? Future research exploring remote people's performance on a battery of basic perceptual and cognitive tasks would help address this question. One possibility is that this figurative “perceptual literacy” may involve growing skill in “imposing one's imagined structure” as in the reversal of ambiguous figures [23], though in this case the structure is derived from one image (photo) and imposed onto another (two-tone). The very act of bringing our knowledge and experience to bear on perception in the way required for cued interpretation and reinterpration of images may be the result of training and experience that is culture-specific.

One prediction of this account is that participants' degree of cultural ubiquity and expertise with decoding visual symbolic materials should relate to their degree of ease and automaticity using visual cues to interpret ambiguous and impoverished images. The present data show that this prediction holds for a special class of two-tone and photo image pairs and for two groups (Pirahã and US) that differ widely in visual symbolic cultural ubiquity, but this relation is at present correlational. And certainly it is not the case that whatever perceptual skills US participants bring to the task are completely absent in the Pirahã (as a group, they did benefit from the presentation of the photo cue, locating the target region on 31.6% of trials). However, if our “perceptual literacy” interpretation is correct, a strong prediction is that levels of reorganization should track with symbolic exposure. Where ubiquity of visual symbolic culture is intermediate between Pirahã and US groups, expertise in decoding such materials should similarly be intermediate.

In their lack of expertise with visual symbols, young U.S. children are similar to the adult Pirahã, whose material culture does not include a writing system, maps, or representational artwork that would entrain such a skill [19]. Enculturation with visual symbolic materials may provide the training required for observers to navigate the dual nature of the two-tone and photo as (1) objects in and of themselves, as well as (2) representations of one another that are mutually informative. In Deloache's research, U.S. children were asked to find a doll hidden in a target room based on the location demonstrated in a symbolic representation of that room. She found surprising and robust failure to use a smaller scale model room to decide where to find a doll hidden in a larger target room, despite accurate memory for the configuration of the model room. The cognitive challenge was to inhibit a prepotent interpretation of the symbolic representation as an entity in and of itself (representation 1), and instead rely on a representation of the correspondence between target and symbol (representation 2). Older more successful children could be impaired if encouraged to play with the objects in the scale model room, strengthening their representation of the room as an entity in and of itself, overriding the representation that mapped the scale model (visual symbolic artifact) to the target room. Conversely, younger children were more successful when the scale model room was placed behind glass, a small change that could help inhibit their representation of the scale model as an entity in and of itself [24].

Simplifying the challenge of dual representation (inhibiting representation 1 and strengthening representation 2) has been shown to aid inexperienced members of a culture (young U.S. children) in using symbolically corresponding visual representations, even before they acquire expertise in ‘reading’ visual symbols such as writing and maps [22]. One memorable method involved convincing children that the scale model room was physically the same entity as the target room due to transformation by “shrinking machine” – thus inhibiting representation 1, removing an interfering representation of the scale model room as a distinct entity. It may be that similar manipulations could enable Pirahã adults to reliably experience photo-cued perceptual reorganization in the absence of a lifelong enculturation with visual symbolic materials. Further research should address the possibility that cultures that provide training in how to ‘read’ visual symbols such as writing and maps can influence the practices of perceptual inference and interpretation required for successful perceptual reorganization.

## Supporting Information

Dataset S1 **Excel sheet containing all data used in the paper.** Raw data for the Pirahã participants and US participants are in sheet and sheet 2, respectively. Analyses by item and by subject are in sheets 3 and 4, respectively. Data for plotting is in sheet 5. Plots are in sheet 6. Statistical tests are in sheet 7.
(XLSX)

File S1 **Supporting figures.** Figure S1, Practice item 1 (Jaguar) at full size. When printed on 11×8.5 inch paper, these images are the same size as the cards used in the experiments. The left image is the original image, and the right image is the transformed (blurred) image. Figure S2, Practice item 2 (Houseboat) at full size. The left image is the original image, and the right image is the transformed (gray-scale) image. Figure S3, Test item 1 (Alligator) at full size. The left image is the original and the right image is the two-tone. Figure S4, Test item 2 (Sloths) at full size. Figure S5, Test item 3 (Hut) at full size. Figure S6, Test item 4 (Older man) at full size. Figure S7, Test item 5 (Squirrel monkey) at full size. Figure S8, Test item 6 (Ocelot) at full size. Figure S9, Test item 7 (Fisherman) at full size. Figure S10, Test item 8 (Howler monkey) at full size. Figure S11, Test item 9 (Toucan) at full size. Figure S12, Test item 10 (Tapir) at full size.
(PDF)
