# Supplementary material for: Cultural Differences in Perceptual Reorganization in US and Pirahã Adults
Source: PLoS One. 2014 Nov 20;9(11):e110225. doi: 10.1371/journal.pone.0110225 (PMC4238998; doi:10.1371/journal.pone.0110225)

Figure S1. Jaguar

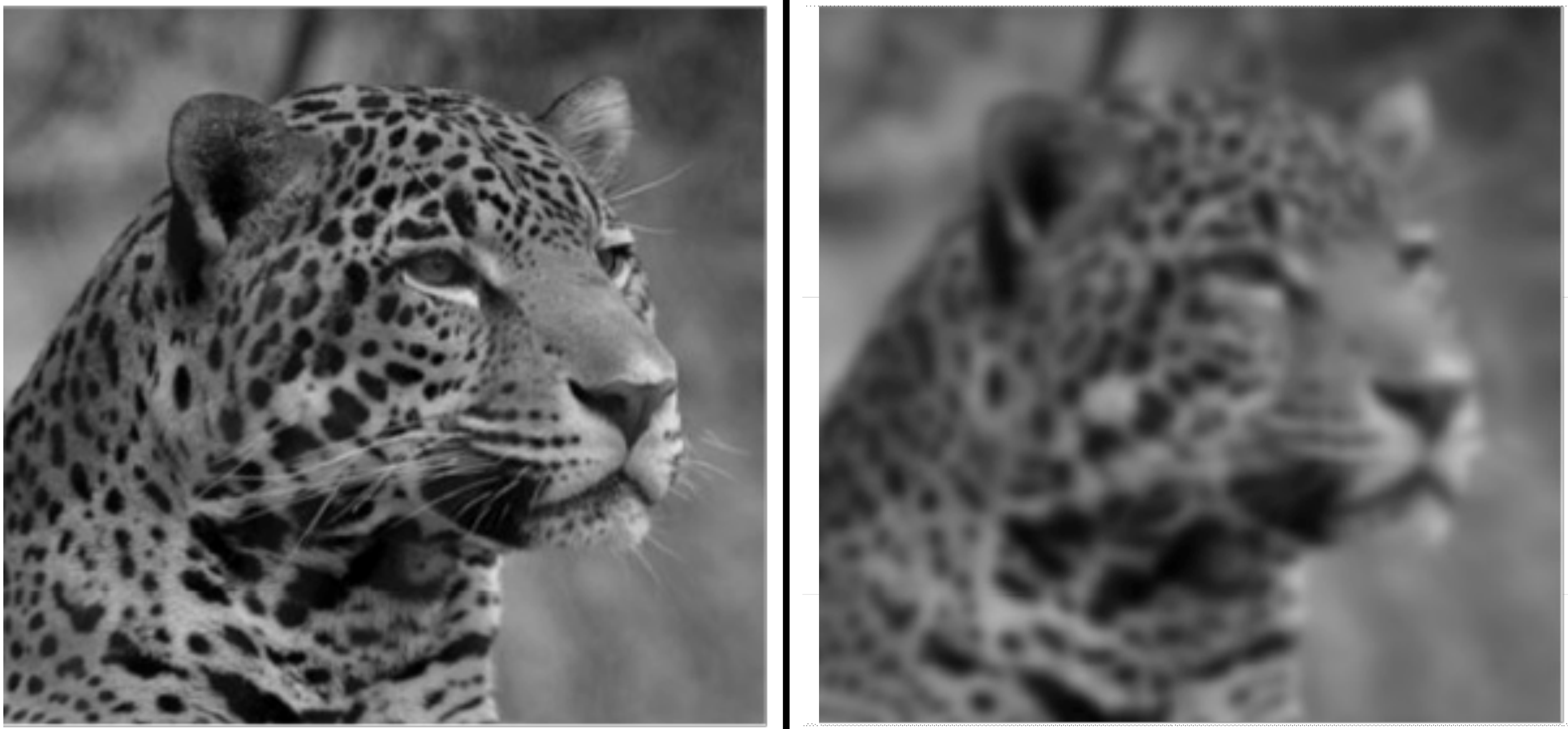

Figure S2. Houseboat

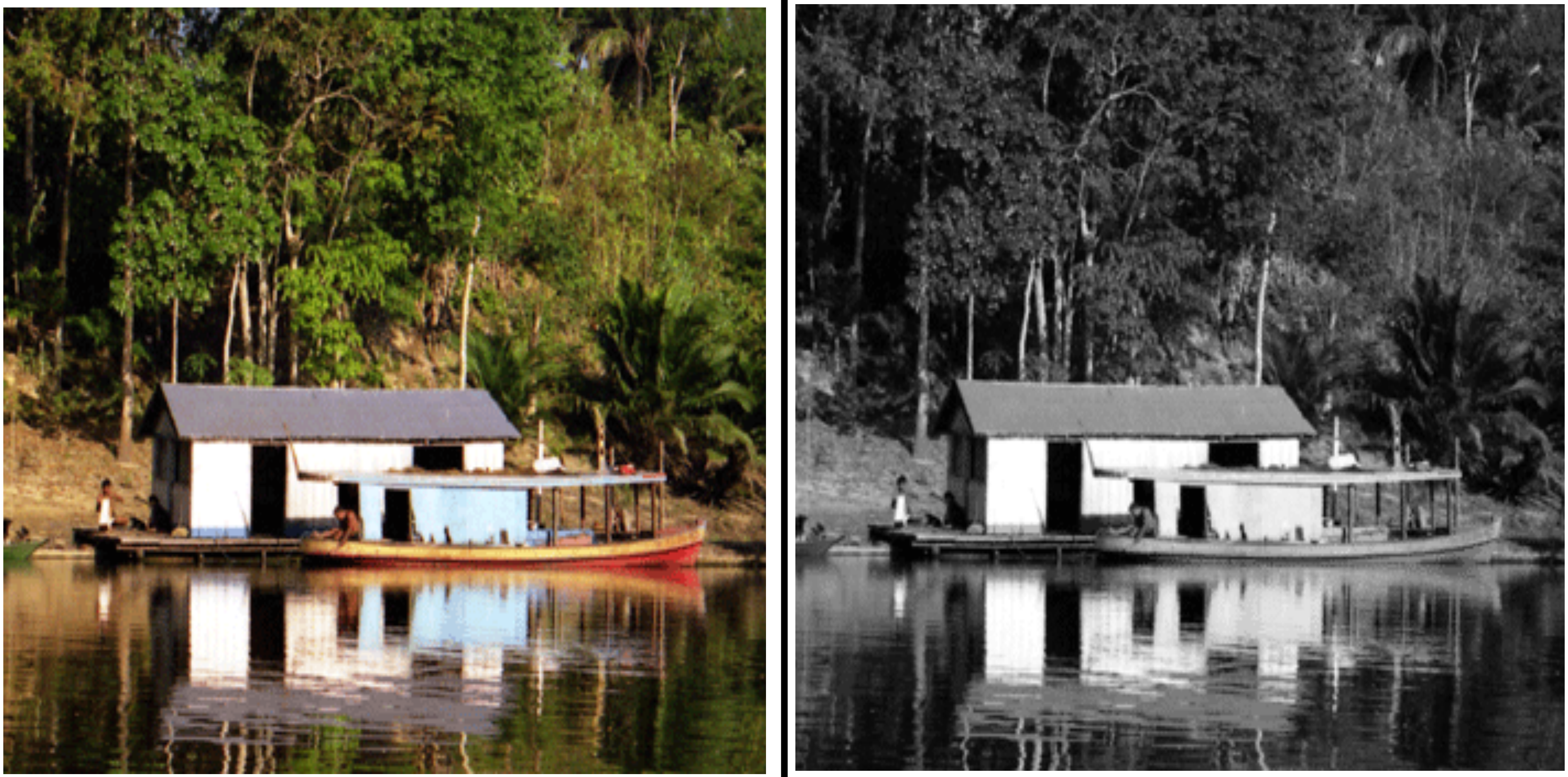

Figure S3. Alligator

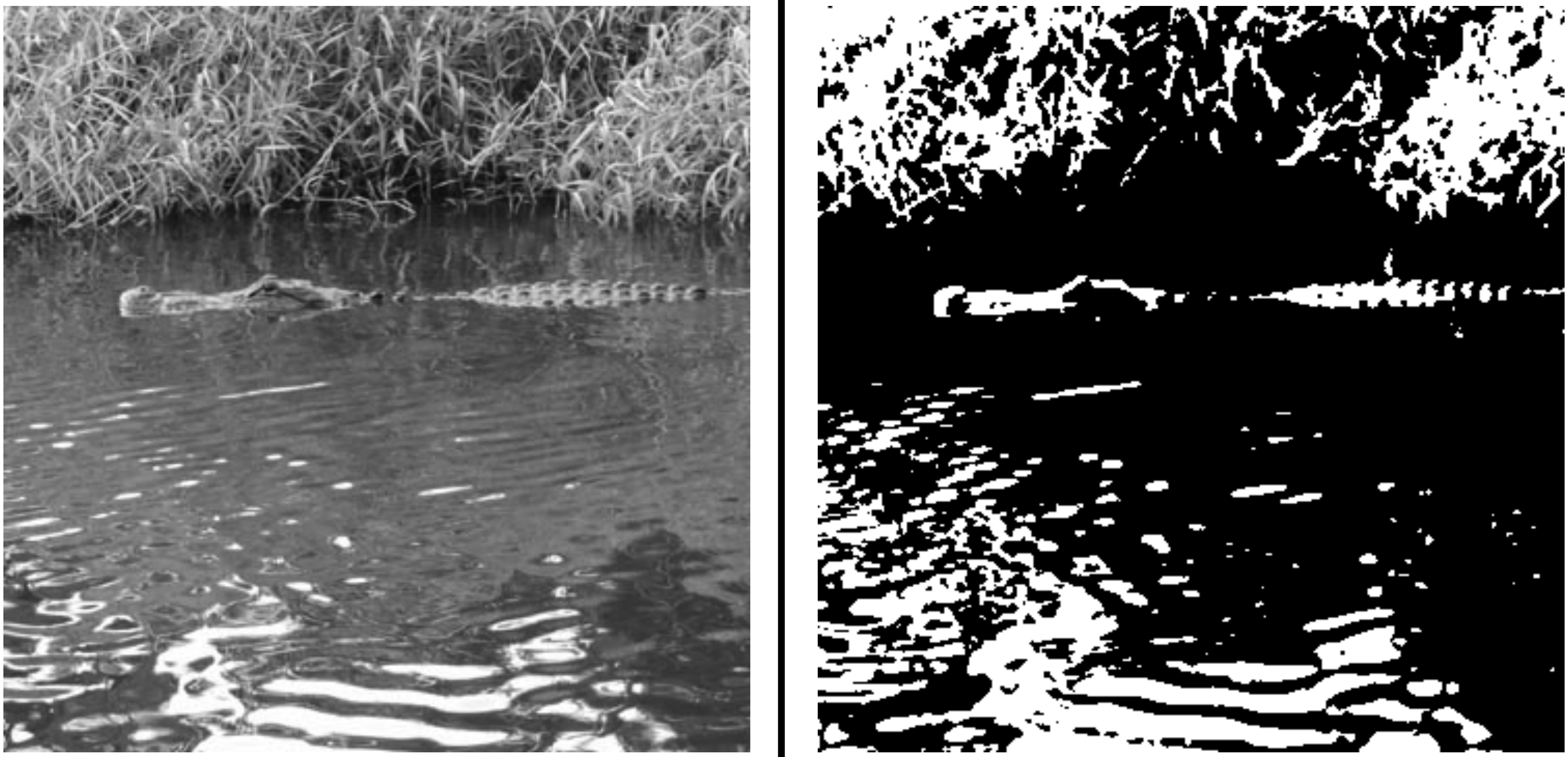

Figure S4. Sloths

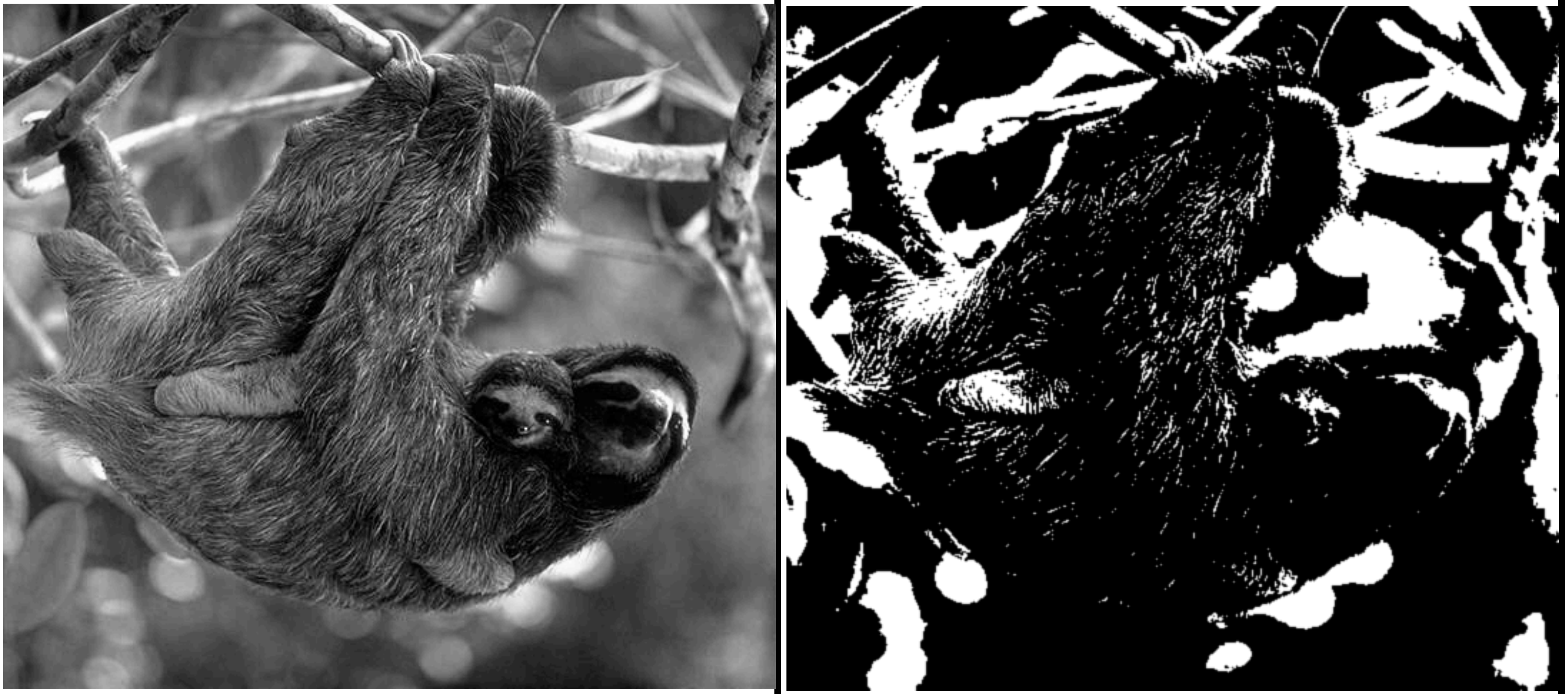

Figure S5. Hut

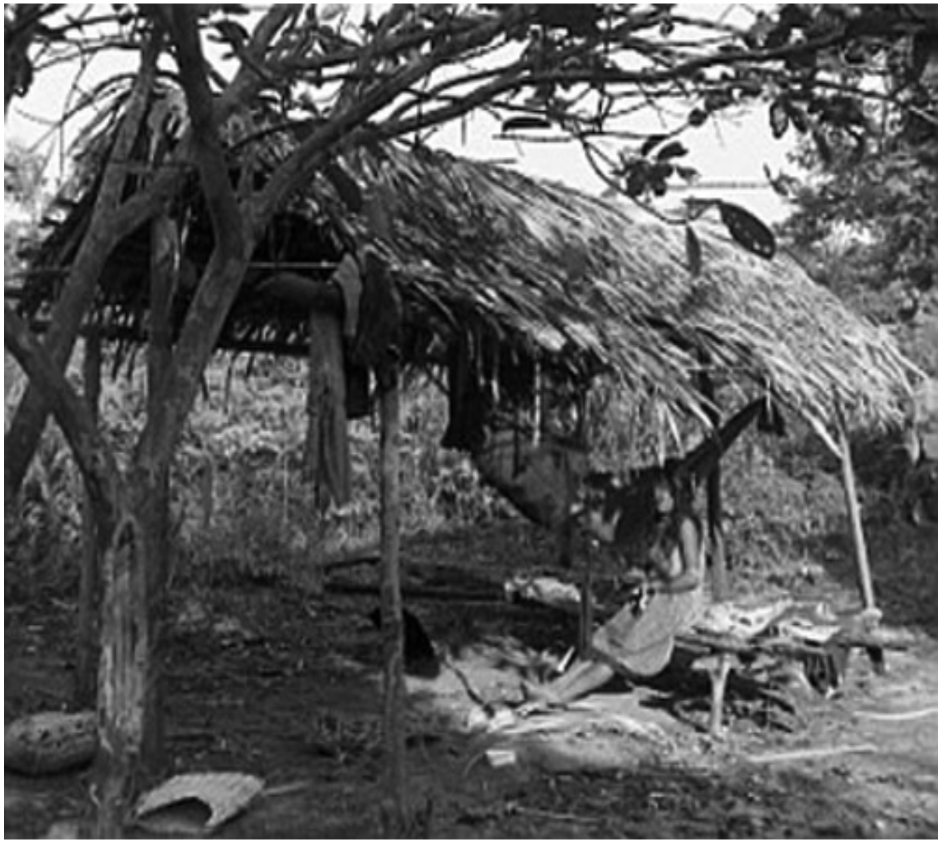

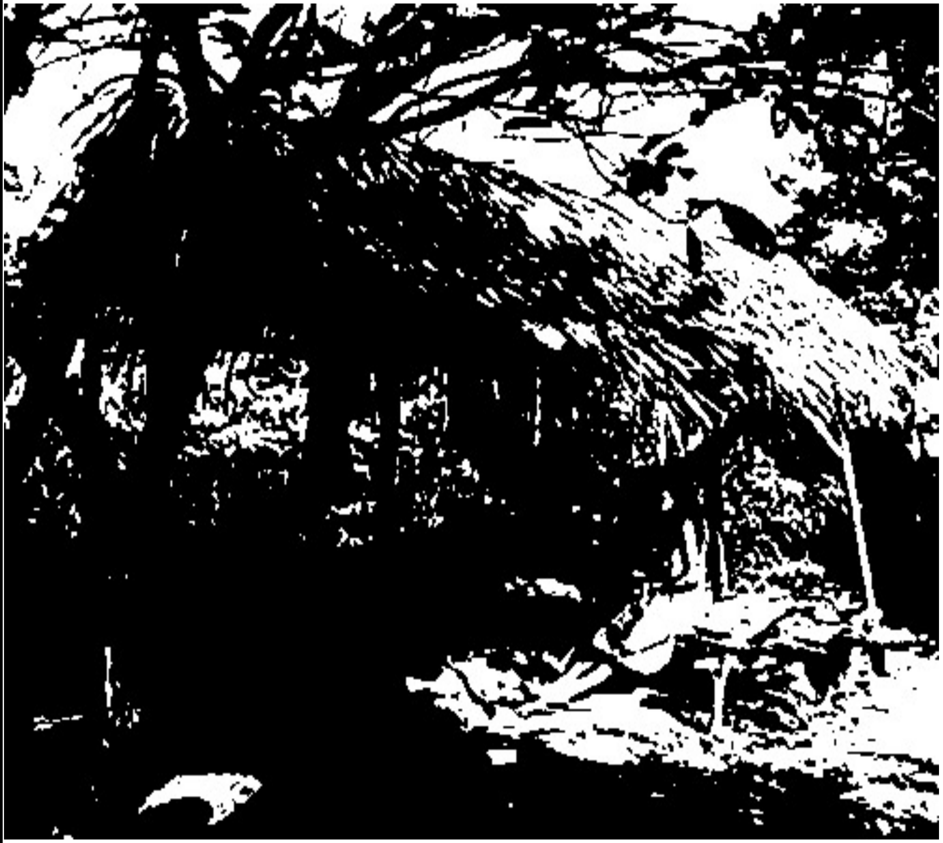

Figure S6. Older Man

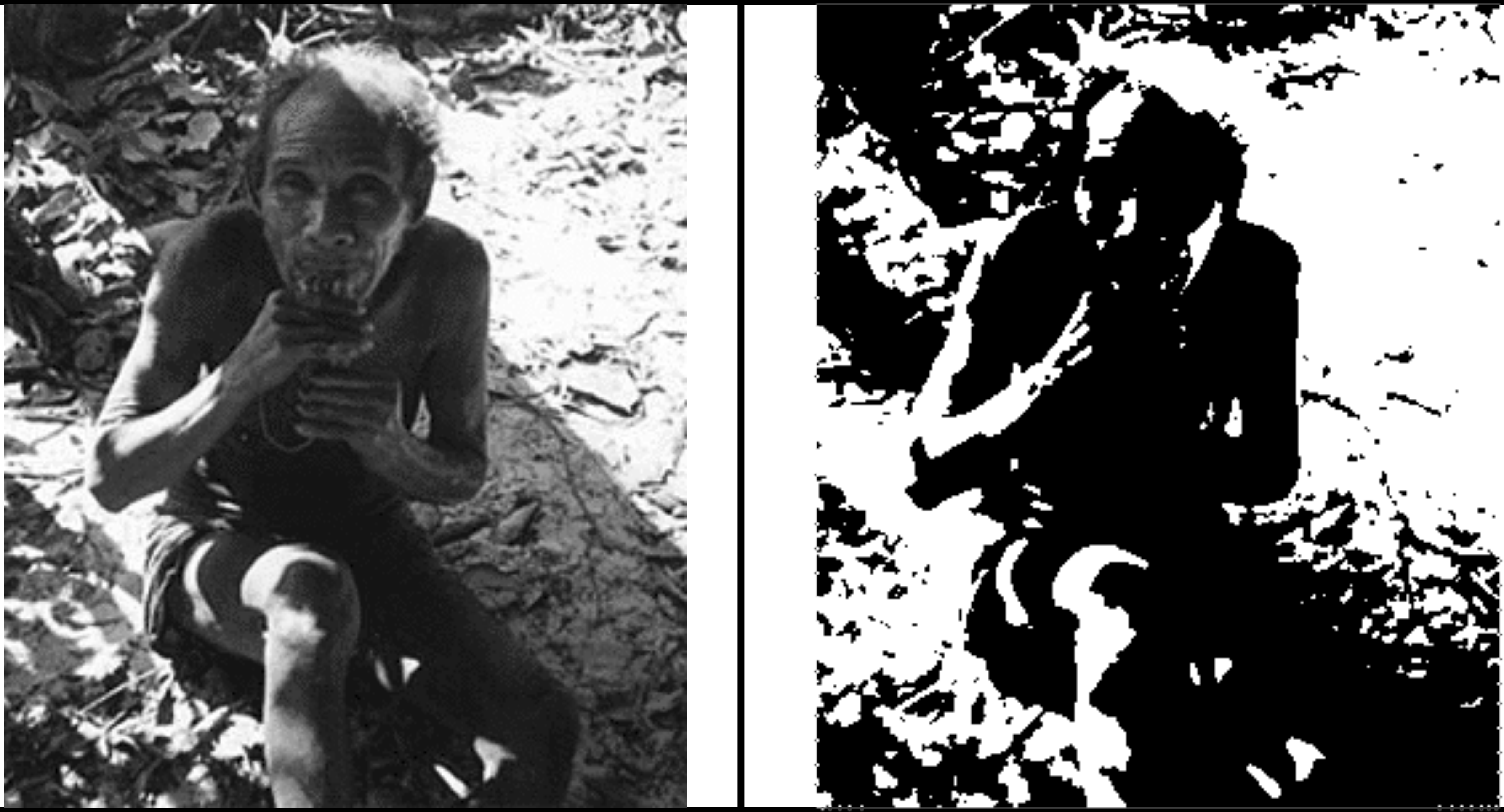

Figure S7. Squirrel Monkey

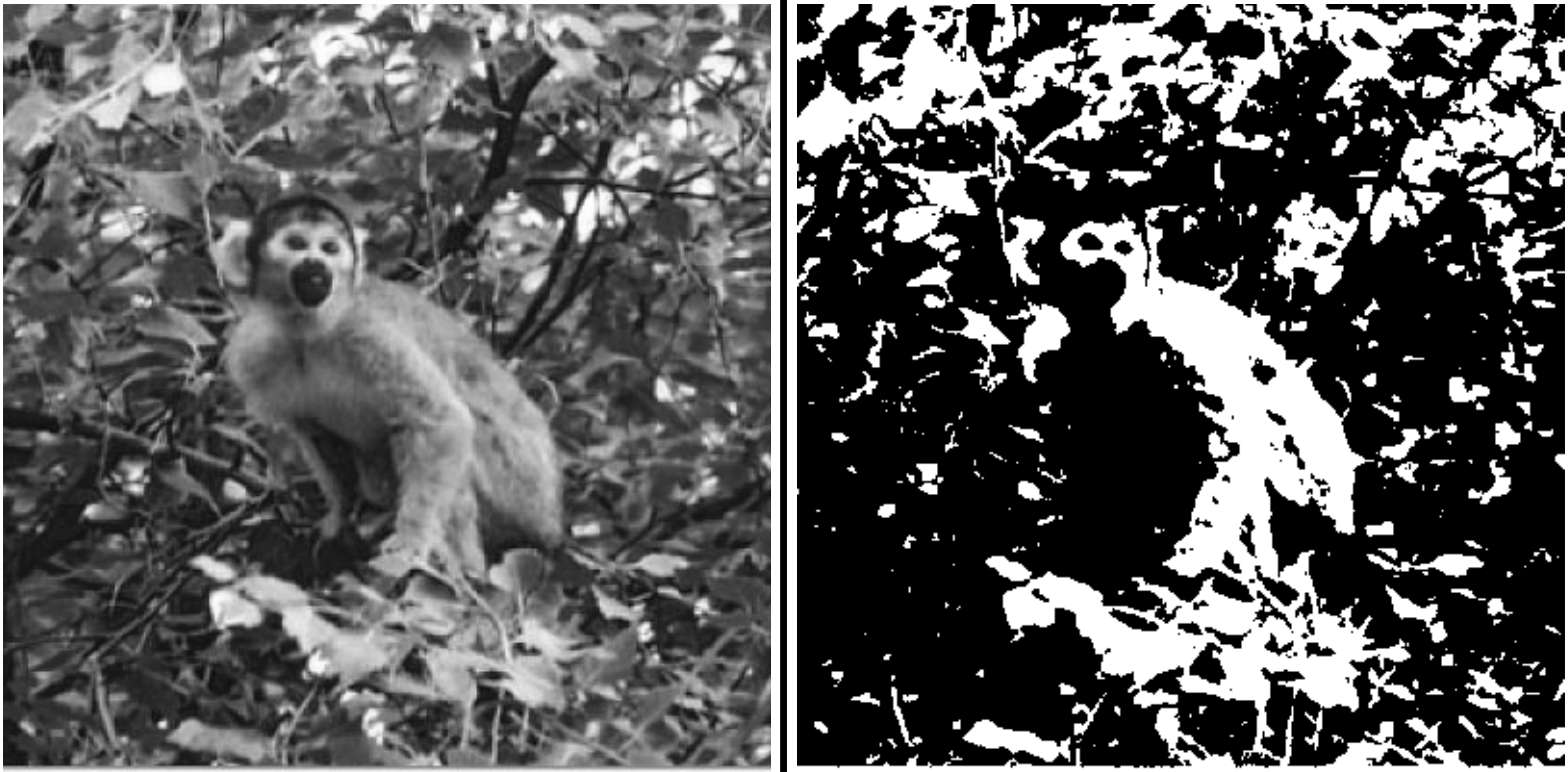

Figure S8. Ocelot

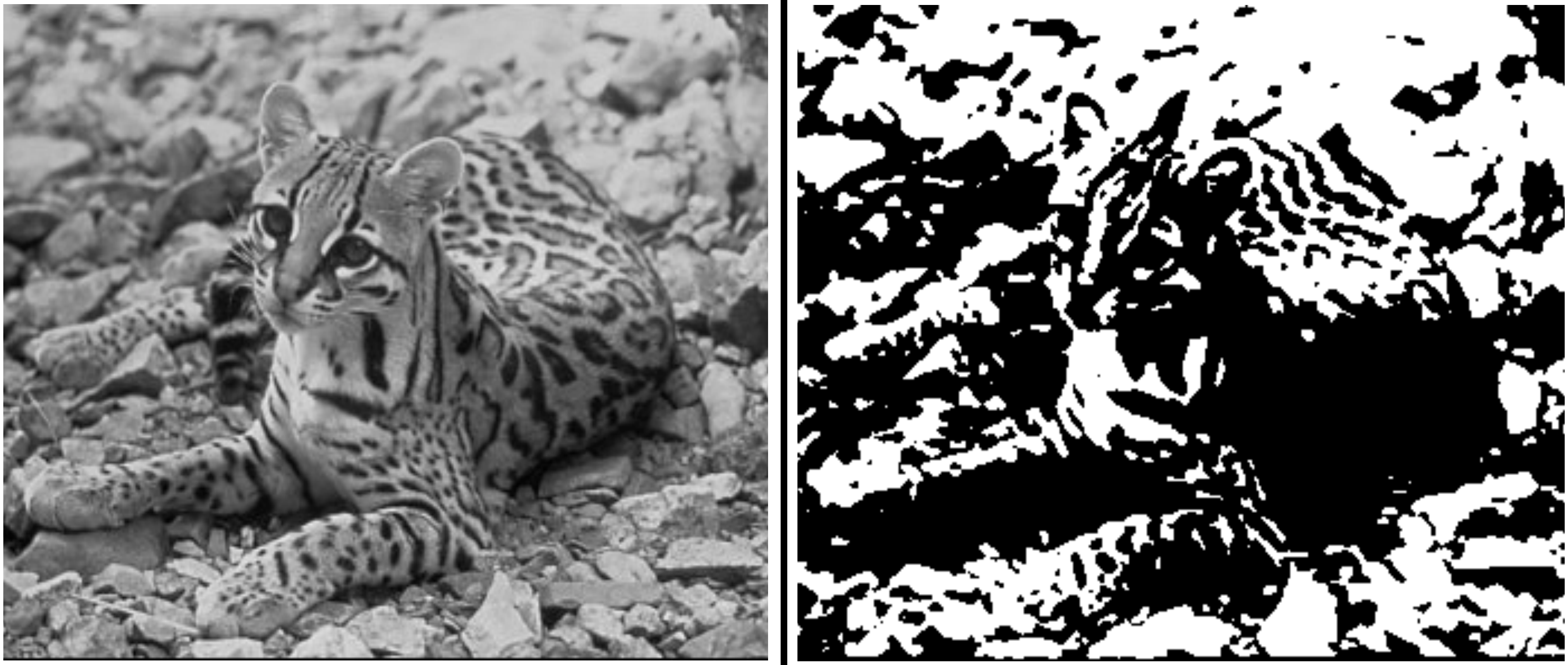

Figure S9. Fisherman

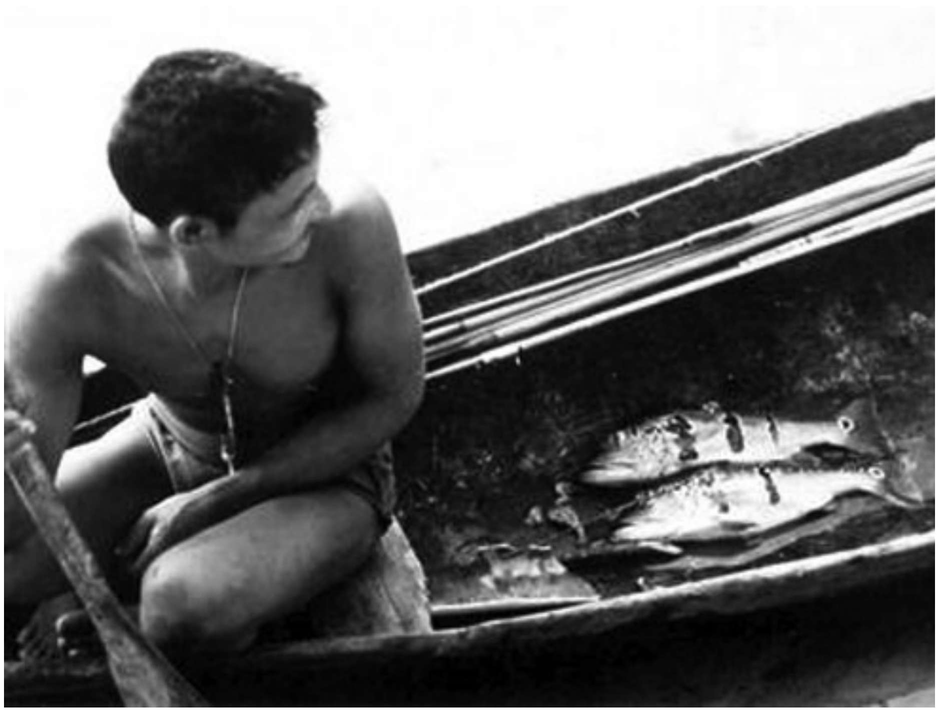

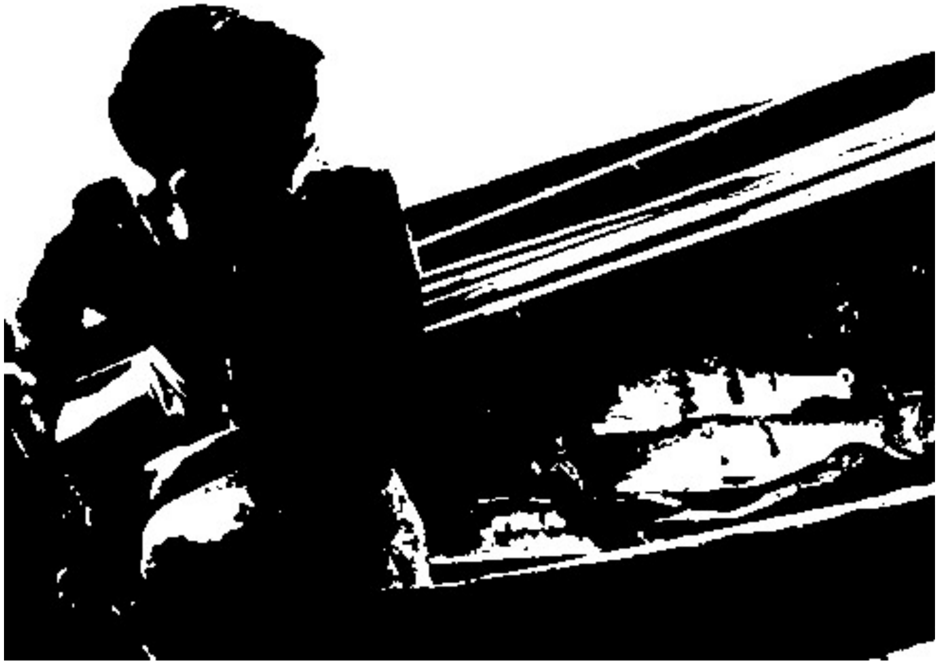

Figure S10. Howler Monkey

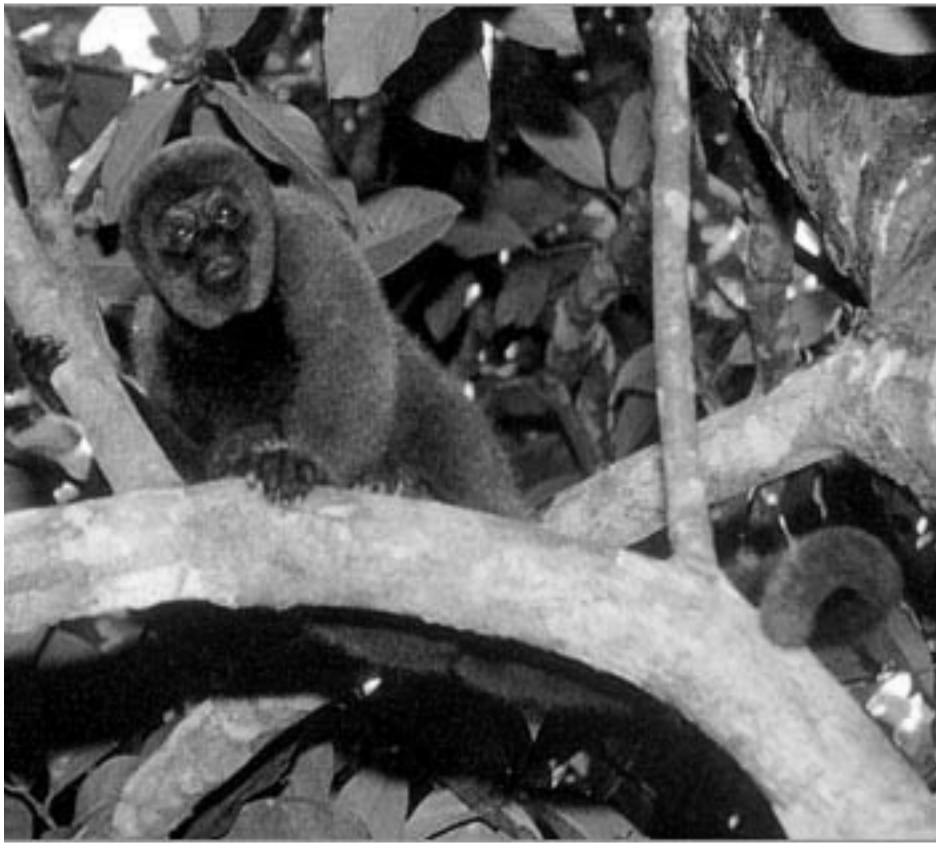

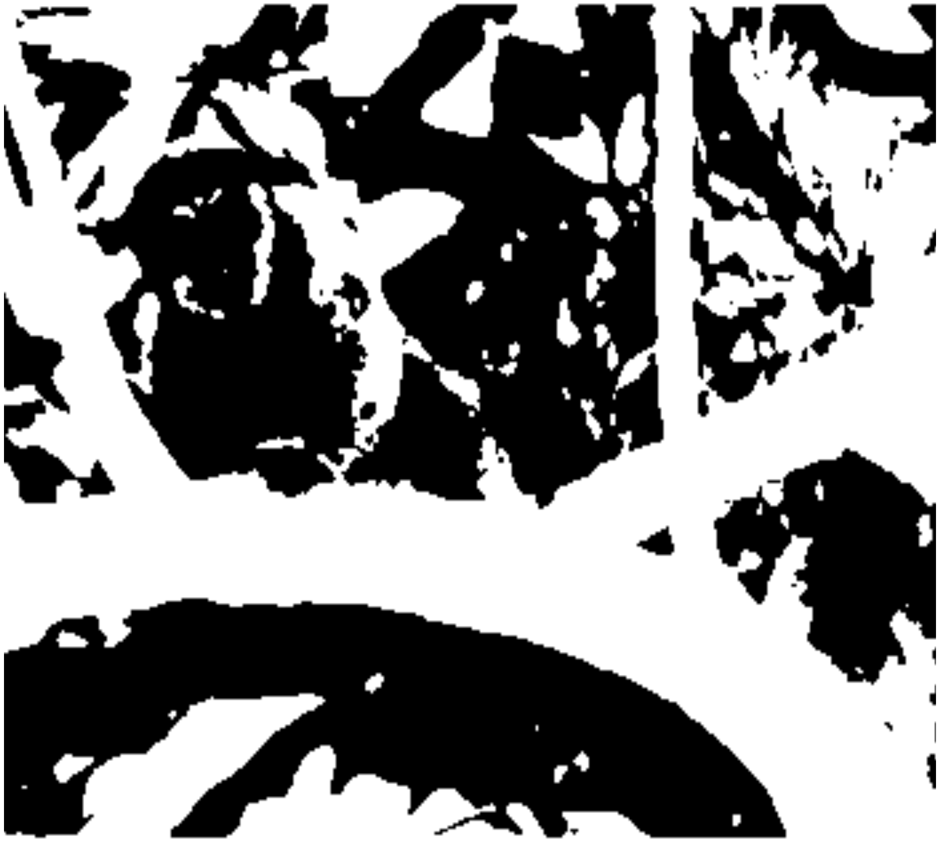

Figure S11. Toucan

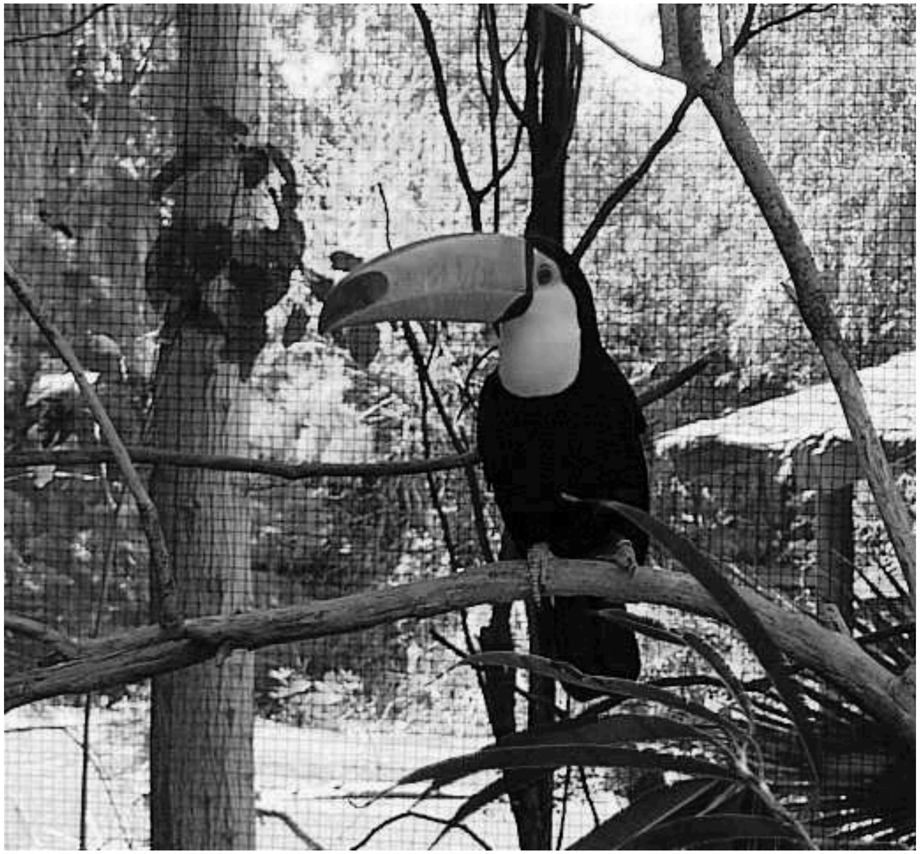

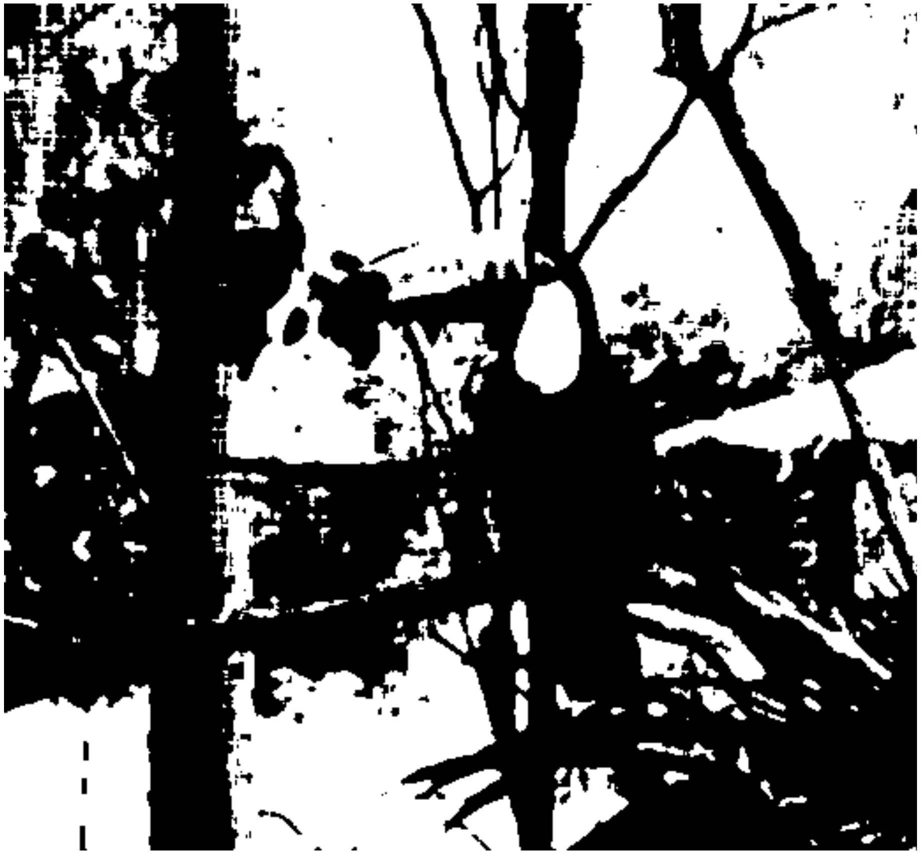

Figure S12. Tapir

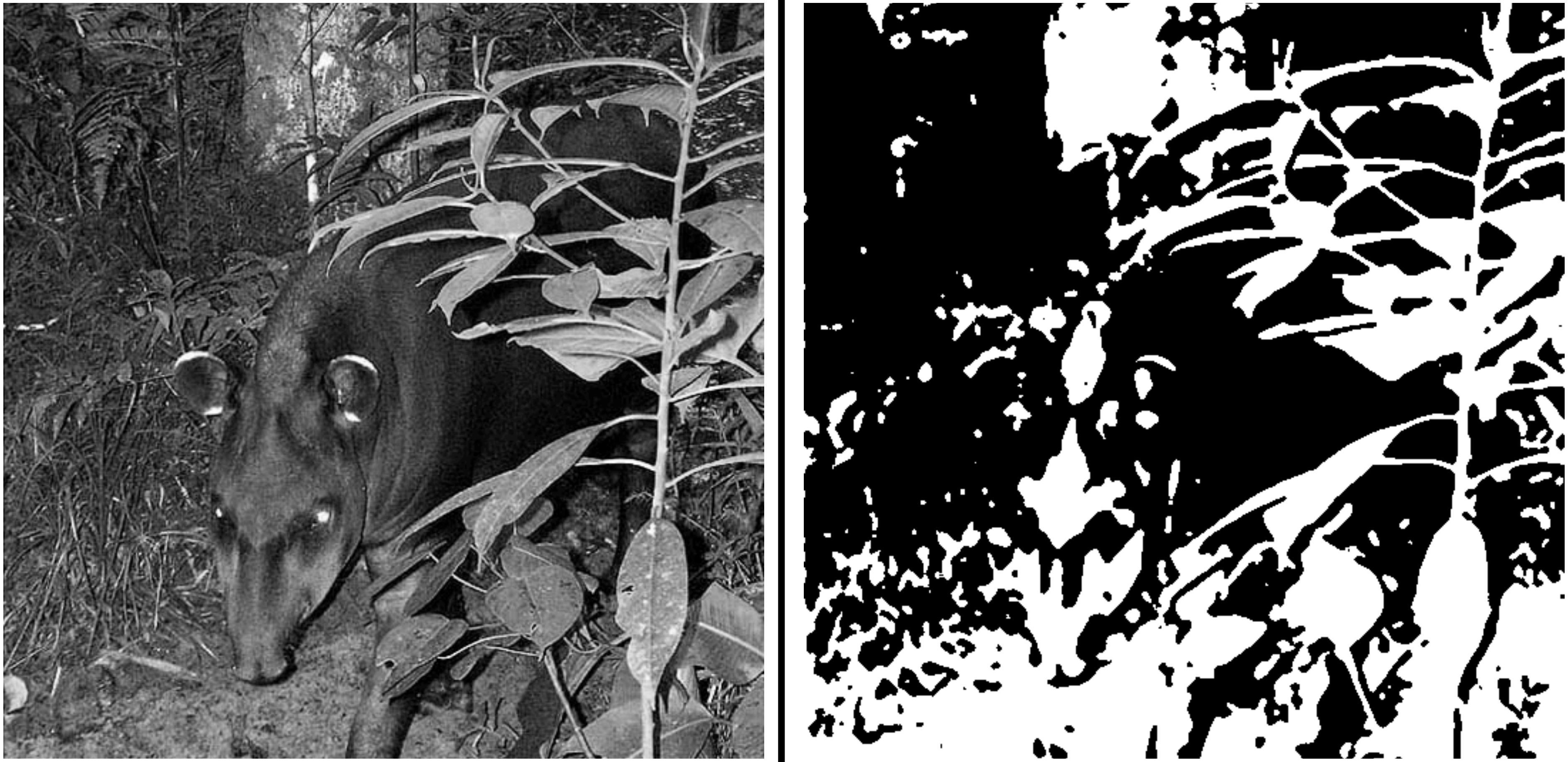

Supplement: File S1 — Supporting figures. Figure S1, Practice item 1 (Jaguar) at full size. When printed on 11×8.5 inch paper, these images are the same size as the cards used in the experiments. The left image is the original image, and the right image is the transformed (blurred) image. Figure S2, Practice item 2 (Houseboat) at full size. The left image is the original image, and the right image is the transformed (gray-scale) image. Figure S3, Test item 1 (Alligator) at full size. The left image is the original and the right image is the two-tone. Figure S4, Test item 2 (Sloths) at full size. Figure S5, Test item 3 (Hut) at full size. Figure S6, Test item 4 (Older man) at full size. Figure S7, Test item 5 (Squirrel monkey) at full size. Figure S8, Test item 6 (Ocelot) at full size. Figure S9, Test item 7 (Fisherman) at full size. Figure S10, Test item 8 (Howler monkey) at full size. Figure S11, Test item 9 (Toucan) at full size. Figure S12, Test item 10 (Tapir) at full size. (PDF) [file pone.0110225.s002.pdf]
